# NEIGHBORHOOD DISADVANTAGE, SOCIAL RELATIONSHIPS, AND COGNITION AMONG MEXICAN-AMERICAN OLDER ADULTS

**DOI:** 10.1093/geroni/igae098.0486

**Published:** 2024-12-31

**Authors:** Kiana Scambray, Angela Gutierrez, Monica Walters, Cindy Tsotsoros, Ketlyne Sol, HwaJung Choi

**Affiliations:** University of Michigan, Ann Arbor, Michigan, United States; Western University of Health Sciences, Pomona, California, United States; University of Michigan, Ann Arbor, Michigan, United States; University of Rhode Island, Kingston, Rhode Island, United States; University of Michigan, Ann Arbor, Michigan, United States; University of Michigan, Ann Arbor, Michigan, United States

## Abstract

Research suggests that Hispanics live in more disadvantaged neighborhoods, and neighborhood disadvantage itself is associated with worse cognition. Few studies have examined whether social factors could buffer this relationship among Hispanic older adults. We examined whether social network size and relationship quality moderates the relationship between neighborhood disadvantage and cognition, and whether these moderations differed by gender in this cross-sectional study. We included 2069 participants (Mean age=81.9; 62% women) from the fifth wave of the Hispanic Established Populations for the Epidemiological Study of the Elderly (H-EPESE). Social network size was defined as the total number of children, relatives, and friends. Relationship quality was defined by how frequently the individual can count on and discuss deep problems with friends/family. Neighborhood disadvantage quartiles (least to most disadvantaged) were created from measures in the National Neighborhood Data Archive after linking with the H-EPESE individual-level data using the census-tract geocode. The Mini Mental State Examination, measuring global cognition, was the outcome. Multiple linear regressions with interaction terms were conducted to assess for moderation, which were repeated across the gender-stratified samples. Relationship quality moderated the association between neighborhood disadvantage and cognition whereas social network size did not. The adverse effect of living in the most disadvantaged neighborhood on cognition was observed among those reporting medium relationship quality (B=-2.94, p<.05). This relationship appeared to be primarily driven by women (B=-5.49, p=.02). These findings suggest that interventions to improve cognition among Hispanic older adults in disadvantaged neighborhoods should consider how relationship quality and gender impact cognition.

